# Exploring the impact of defect energy levels in CdTe/Si dual-junction solar cells using wxAMPS

**DOI:** 10.1038/s41598-024-55616-2

**Published:** 2024-02-27

**Authors:** Mustapha Isah, Camellia Doroody, Kazi Sajedur Rahman, Mohd Nazri Abd Rahman, Adamu Ahmed Goje, Manzoore Elahi M. Soudagar, Tiong Sieh Kiong, Nabisab Mujawar Mubarak, Ahmad Wafi Mahmood Zuhdi

**Affiliations:** 1 College of Engineering, Universiti Tenaga Nasional, Jalan IKRAM-UNITEN, 43000 Kajang, Selangor Malaysia; 2 Department of Physics, Kaduna State University, PMB 2339, Tafawa Balewa Way, Kaduna, Kaduna State Nigeria; 3 Institute of Sustainable Energy (ISE), Universiti Tenaga Nasional, Jalan IKRAM-UNITEN, 43000 Kajang, Selangor Malaysia; 4 Solar Energy Research Institute, Universiti Kebangsaan Malaysia, 43600 Bangi, Selangor Malaysia; 5 Institute of Power Engineering, Universiti Tenaga Nasional, Jalan IKRAM-UNITEN, 43000 Kajang, Selangor Malaysia; 6 Department of Science Laboratory Technology, Federal Polytechnic Damaturu, Damaturu, Yobe State Nigeria; 7 Department of Mechanical Engineering, Graphic Era (Deemed to be University), Dehradun, Uttarakhand 248002 India; 8 Petroleum and Chemical Engineering, Faculty of Engineering, Universiti Teknologi Brunei, Bandar Seri Begawan, BE1410 Brunei Darussalam; 9 Department of Chemistry, School of Chemical Engineering and Physical Sciences, Lovely Professional University, Jalandhar, Punjab India

**Keywords:** Energy, Defect density, Trap levels, CdTe/Si tandem solar cell, Multijunction solar cells, wxAMPS, Energy science and technology, Engineering, Materials science, Mathematics and computing

## Abstract

A numerical analysis of a CdTe/Si dual-junction solar cell in terms of defect density introduced at various defect energy levels in the absorber layer is provided. The impact of defect concentration is analyzed against the thickness of the CdTe layer, and variation of the top and bottom cell bandgaps is studied. The results show that CdTe thin film with defects density between 10^14^ and 10^15^ cm^−3^ is acceptable for the top cell of the designed dual-junction solar cell. The variations of the defect concentrations against the thickness of the CdTe layer indicate that the open circuit voltage, short circuit current density, and efficiency (ƞ) are more affected by the defect density at higher CdTe thickness. In contrast, the Fill factor is mainly affected by the defect density, regardless of the thin film’s thickness. An acceptable defect density of up to 10^15^ cm^−3^ at a CdTe thickness of 300 nm was obtained from this work. The bandgap variation shows optimal results for a CdTe with bandgaps ranging from 1.45 to 1.7 eV in tandem with a Si bandgap of about 1.1 eV. This study highlights the significance of tailoring defect density at different energy levels to realize viable CdTe/Si dual junction tandem solar cells. It also demonstrates how the impact of defect concentration changes with the thickness of the solar cell absorber layer.

## Introduction

Multijunction solar cells are the rising generation of photovoltaic devices that are already breaking the efficiency limit of the present highly researched single-junction solar cells^1–3^. Both CdTe and Si stand as prominent materials in the solar cell market, benefitting from well-established processing techniques. The advantageous attributes of CdTe, such as its high absorption coefficient reaching about 10^4^ cm^−1^ due to its direct band gap, make it particularly proficient at absorbing photons incident within a mere 1 μm of its thickness. Doping CdTe with elements like Zn, Mg, and Se has proven effective in elevating its bandgap to over 1.7 eV. This enhancement aligns closely with silicon (Si), which boasts a band gap of approximately 1.12 eV when employed in a tandem configuration. The strategic pairing of these devices offers the potential to exceed the theoretical efficiency limit of around 34% for single-junction solar cells. There is also enormous potential to push solar cell technology beyond current boundaries by enhancing the compatibility and proven techniques of CdTe and Si, ushering in a new age of increased efficiency and performance. Few studies have been conducted on integrating CdTe thin film solar cells and their alloys with Si solar cells as tandem photovoltaic devices. Most of these studies initially design and optimize the CdTe/Si device structure using different numerical simulation software such as AMPS-1D, wxAMPS, SCAPS, SILVACO, Sentaurus Device, etc. In a study carried out by Enam et al., 2017, where they designed CdTe/Si using a heavily doped p++ CdTe/n++ Si tunnel diode linking the top and the bottom cells by simulation, they obtained an optimum efficiency of 28.45% after optimizing the absorber layers, carrier concentrations, and operating temperature^4^. In another work, Tamboli et al.^5^ used device modeling and bottom-up techno-economic analysis to show that CdTe/Si tandem solar cells have the potential to provide over 30% with a cost comparable to or lower than either single junction Si or CdTe solar cells, especially where space is a bottleneck. Zhou et al.^6^, used SCAPS numerical simulation software to design a CdTe/Si composite absorption layer to reduce the mismatch between the CdTe and Si interface. Their result indicates that a thinner top subcell and a thicker bottom cell show higher efficiency^6^. They also showed how the defective states at the interface of CdTe and Si degrade the device performance in CdTe/Si solar cells. Their final optimization of the CdTe/Si tandem device resulted in a maximum efficiency of 28.36%. However, a study on the defect’s concentrations in a CdTe/Si tandem device is yet to be properly carried out. This is needed to check all parameters that can affect the performance of this device when fully developed. Hence, this study focuses on the effect of various defect concentrations at different defect energy levels on the output parameters of a proposed CdTe/Si dual-junction photovoltaic device. Defects like dislocations, interfaces, and grain boundaries can form recombination centres for electron–hole pairs in solar cells^7^. This is a major limiting factor in reaching higher efficiency.

As a polycrystalline absorbing material, CdTe thin film exhibits many grain boundaries and inter-grain dislocations^8^. Defect mapping for CdTe material shows high recombination at 0.448 eV, 0.383 eV, 0.366 eV, and 0.341 eV located within the bandgap of CdTe^9^. Other works revealed that dislocations in the CdTe layer give a shallow to mid-level of around 0.17 eV or a deep level of about 0.7 eV below the conduction band^10,11^. These trap centers or defects are the major contributors that limit the Voc of CdTe solar cells to around 600 mV instead of the theoretical value of 1.2 V. The most optimized laboratory CdTe photovoltaic device still has its Voc around 0.8872 eV, just about 59.15% obtainable from its theoretical bandgap^12^. In the CdTe/Si heterojunction solar cell, the CdS/CdTe interface and the CdTe absorber layer are the regions where most defects can occur due to the thin film deposition techniques used and the polycrystalline nature of CdTe compared to the highly controlled fabrication methods and crystalline nature of Si solar cells^13^. The common CdTe thin film deposition techniques are close-spaced sublimation (CSS), sputtering, electrodeposition, chemical spray pyrolysis, thermal evaporation, pulsed laser deposition, etc., which are usually not defect-free processes^14–21^. In addition, in the proposed CdTe/Si tandem device, many lattice imperfections in the CdTe layer are likely due to the lattice and thermal mismatches between CdTe or its alloys with Si^7,22^. Therefore, this work is tailored to understand further the effect of defect densities at various defect energy levels that can be present in the top cell and CdTe absorber layer and how they can affect the performance of the proposed tandem device. In this study, numerical modeling using the wxAMPS simulation software is deployed to introduce defects in various densities at different energy levels within the bandgap of CdTe. Also, the impact of CdTe layer thinning to match the currents of the top cell and that of the bottom cell with various defect densities is studied. A validation of bandgap combinations for the top CdTe and Si bottom absorber layers is also carried out.

## Simulation Method

**Figure 1:**
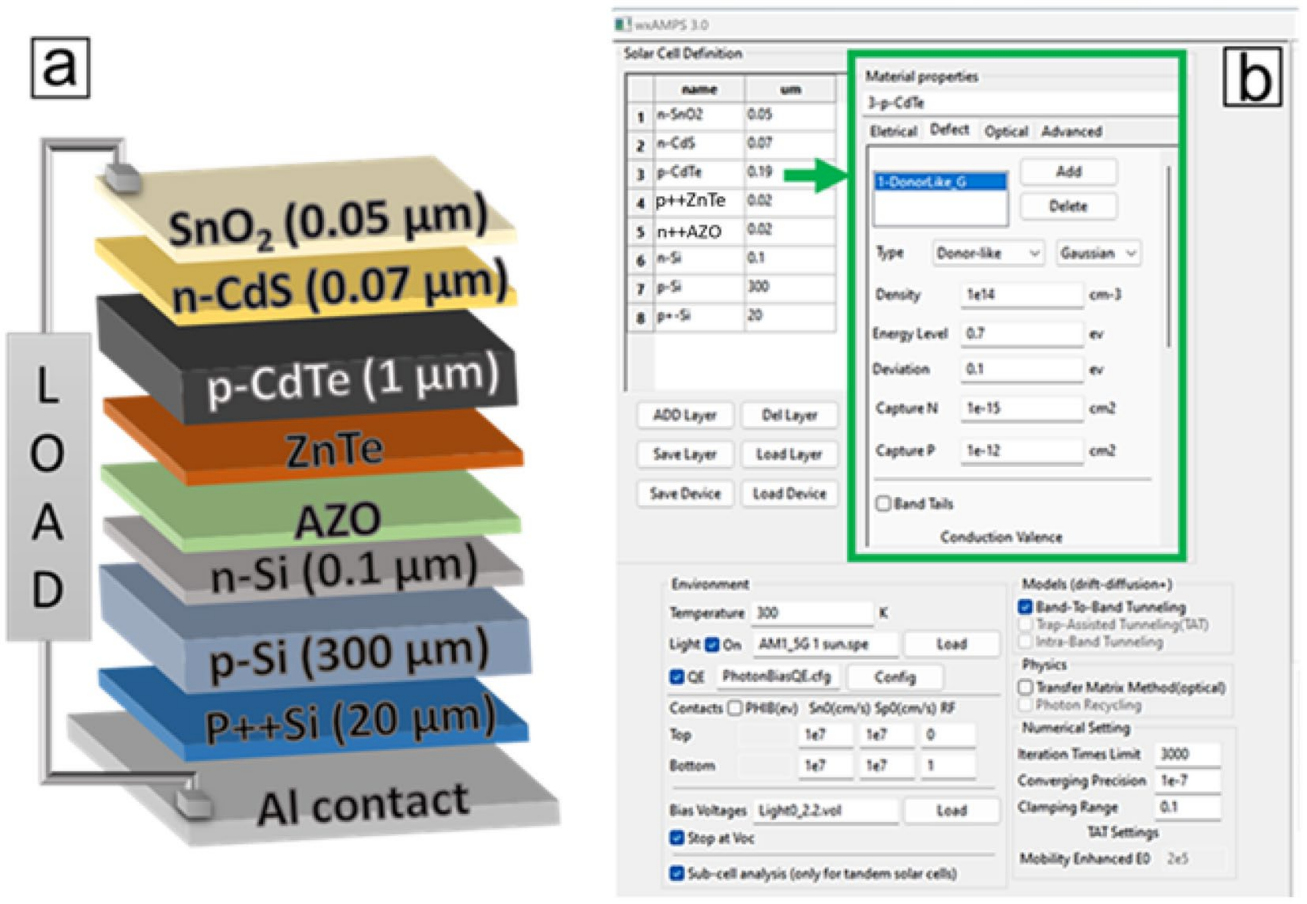
(**a**) Schematic of CdTe/Si tandem stack (**b**) wxAMPS defect definition interface.

wxAMPS 3.0 numerical simulation software is used in this work to simulate the proposed structure. It is a one-dimensional, open-source simulation software for simulating tandem or multijunction solar cells^23^. wxAMPS is built based on the drift–diffusion models for predicting the current–voltage values. It incorporates features such as allowing carrier tunneling from one band to another, carriers tunneling via traps, and intra-band tunneling. These features are crucial in modeling carrier transport in multijunction photovoltaic devices^24^. The tunneling from one band to another band is of two categories. There is local and non-local band-to-band tunneling. The non-local tunnelling is mostly used in analyzing the tunnel junction used in connecting different cells in multijunction solar cells^25^. wxAMPS 3.0 also incorporates tunnelling-improved recombination and field-improved carriers mobilities as given by Eq. (1) to provide a more realistic calculation for a multijunction device as proposed in this work.

1 $$R = \frac{{N_{t} V_{th} \sigma_{n} \left( { 1 + {\Gamma }_{n} } \right)\left( { 1 + {\Gamma }_{p} } \right) \sigma_{p} \left( {np - n_{i}^{2} } \right)}}{{ \sigma_{n} \left( { 1 + {\Gamma }_{n} } \right)\left( {n + n_{t} } \right) + \sigma_{p} \left( { 1 + {\Gamma }_{p} } \right)\left( {p + p_{t} } \right)}}$$

where R represents the recombination rate, ${N}_{t}$ is the variable for defect density $, {V}_{th}$ stands for thermal velocity $,$ $n,$ and $p$ are the carrier concentrations of electrons and holes,${n}_{i}$ is the intrinsic carrier density and ${\sigma }_{n}$ and ${\sigma }_{p}$ symbolise the capture cross-sections for electrons and holes, respectively, ${\Gamma }_{n}$ and ${\Gamma }_{p}$ are the field-driven tunnelling functions that give the contributions of trap-assisted tunnelling to recombination and trap occupation^26^. The mobilities of electrons and holes are also improved as a function of the electric field, which drives the tunnelling transport in high electric field regions^24,27^. Another important tool built in the wxAMPS 3.0 software is the tool for subcell analysis that allows each solar cell in the tandem device to be analysed and optimised individually^28^. In addition, wxAMPS is incorporated with an interface for defect analysis, which is utilised in this study to simulate various defect densities at different defect energy levels within the bandgap of the CdTe absorber layer. wxAMPS has proven to be an effective software in designing and optimising multijunction solar cells^29,30^. The designed two-junction (2 J) CdTe/Si heterostructure is of the form SnO_2_/n-CdS/p-CdTe/p++ ZnTe top cell connected to n++ AZO/n-Si/p-Si/p+-Si/Al bottom cell which is illustrated in Fig. 1. Table 1 gives the parameters used in the simulations.

**Table 1:** Simulation parameters.

Parameters	n-SnO_2_	n-CdS	p+ CdTe	p++ ZnTe	n++ AZO	n-Si	p-Si	p++ Si
Thickness (µm)	0.05	0.07	0.1–1.4	0.02	0.02	0.1	300	20
Electric Permittivity	9.0	10	9.4	9.67	10	11.9	11.9	11.9
Bandgap (eV)	3.40	2.42	1.5	2.26	3.4	1.12	1.12	1.12
Electron affinity (eV)	4.20	4.50	4.28	3.50	3.8	4.05	4.05	4.05
Conduction band effective density of states Nc (cm^−3^)	2.2 × 10^18^	2.2 × 10^18^	7.5 × 10^17^	7.0 × 10^16^	1.5 × 10^18^	2.8 × 10^19^	2.8 × 10^19^	2.8 × 10^19^
Valence band effective density of states Nv (cm^−3^)	1.8 × 10^19^	1.8 × 10^19^	1.8 × 10^19^	2.0 × 10^20^	1.8 × 10^19^	1.04 × 10^19^	1.04 × 10^19^	1.04 × 10^19^
Electron mobility Un (cm^2^/Vs)	100	100	320	330	100	1350	1350	1350
Hole mobility up (cm^2^/Vs)	25	25	40	80	50	450	450	450
Donor concentration Nd (cm^−3^)	1 × 10^17^	1 × 10^17^			1 × 10^19^	1.0 × 10^16^		
Acceptor concentration Na (cm^−3^)			1.0 × 10^16^	1.0 × 10^20^			1.0 × 10^16^	1.0 × 10^19^
Defect density (cm^−3^)		1 × 10^14^	10^10^ to 10^16^					
Capture cross section for electrons (cm^2^)		1 × 10^−13^	1 × 10^−15^					
Capture cross section for holes (cm^2^)		1 × 10^−15^	1 × 10^−12^					
Defect energy levels (eV)		1.21	− 0.1 to 0.8					
Energy deviation (eV)		0.1	0.1					

## Results and discussion

### Top cell optimization

**Figure 2:**
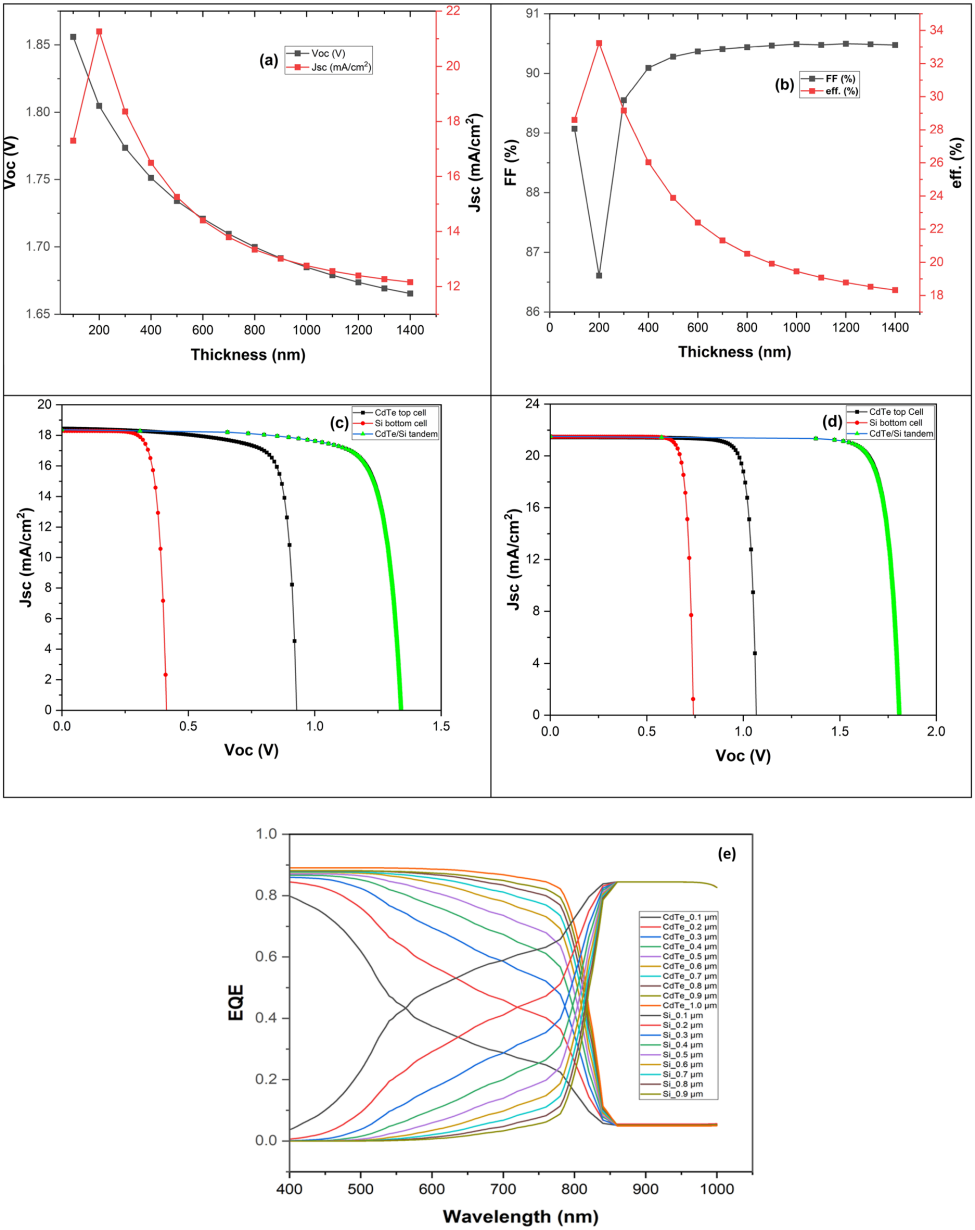
Variation of the CdTe top cell absorber layer thickness with (**a**) Voc and Jsc, (**b**) fill factor and efficiency, (**c**) and (**d**) the matched top and bottom sub-cells I-V curves for the baseline and optimized CdTe/Si tandem designs. (**e**) external quantum efficiency variation with CdTe thickness.

Our proposed CdTe/Si dual-junction solar cell comprises a basic CdTe solar cell structure (n-CdS/p-CdTe) as the top cell that is connected to a Si bottom cell (n-Si/p-Si) via a p++ ZnTe/n++ AZO tunnel diode as shown in Fig. 1. The choice of an n-type Si emitter layer on a p-type Si absorber layer despite the latter having lower diffusion length is to match the n/p tandem polarity of the proposed tandem solar cell as p-type CdTe solar cell is more established in practice than n-type. CdTe has an optical bandgap of 1.5 eV, and this implies that CdTe will be transparent to photons having a wavelength ranging between 825 and 1100 nm but will be absorbed by Si due to its bandgap of 1.12 eV as a bottom subcell with CdTe as top sub-cell. One fundamental way to harness optimum Jsc from a tandem device is to ensure that the Jsc generated by the top and bottom subcells are matched by optimizing the top cell thickness. After fixing other simulation parameters, as shown in Table 1, thickness variations between 100 nm and 1400 nm were used for the CdTe top layer at a fixed Si bottom cell thickness of 300 µm to match their current densities. Figure 2a indicates that Jsc and Voc initially reduced rapidly with an increasing absorber layer thickness and reached their top values at around 200 nm. Figure 2b indicates that the FF increases with increasing thickness. At the same time, the efficiency drops sharply as the thickness increases and becomes more saturated as it reaches around 1.2 µm of CdTe thickness. An interception between the FF and the efficiency occurred at around 300 nm of the CdTe thickness. Conceptually, efficiency drops while FF increases if thickness of the absorber layer increases beyond the diffusion length of the generated carriers. Another factor that plays a role in decreasing the efficiency as FF increases as in this case is, as the CdTe top layer gets thicker, less photons are reaching the bottom cell, and this drastically reduces the Jsc of the bottom cell. It has been shown that final Jsc of the tandem device is always limited by the lower bottom cell current this is why it is important to optimise the thickness of the top layer to match the top and bottom cells currents^31–33^. The optimum current matching was obtained at around 200 nm CdTe thickness, in tandem with the result obtained in^6^. Figures 2c,d show the current matchings of the baseline (without tunnel diode) and the optimized CdTe/Si tandem designs. The Jsc curves in the baseline bend more than the optimized curves, possibly due to more series resistance arising from the absence of an ohmic tunnel diode in the baseline design. It can also be seen that more Jsc and Voc are obtained in the optimized design, this could be attributed to the reduction of recombination at the interface of the top and bottom cells after inserting the tunnel diode. The external quantum efficiency (EQE) plot in Fig. 2e highlights how the variation in the thickness of the CdTe layer changes with the quantum efficiency of the top and bottom cells of the simulated device. The thickness has to be carefully optimized and chosen to not lose photocurrent due to a thinner absorber layer or have more carrier recombination due to the thickness being thicker than the carrier diffusion length^34,35^. The EQE plot reveals the tradeoffs between the photons absorbed by the top and bottom absorbing materials due to the CdTe thickness variations. This ensures an equal amount of current is generated between the two cells. This is done to eliminate the effect of current mismatch, which usually results in poor performance by tandem solar cells.

### p++ ZnTe/n++ AZO tunnelling junction

**Figure 3:**
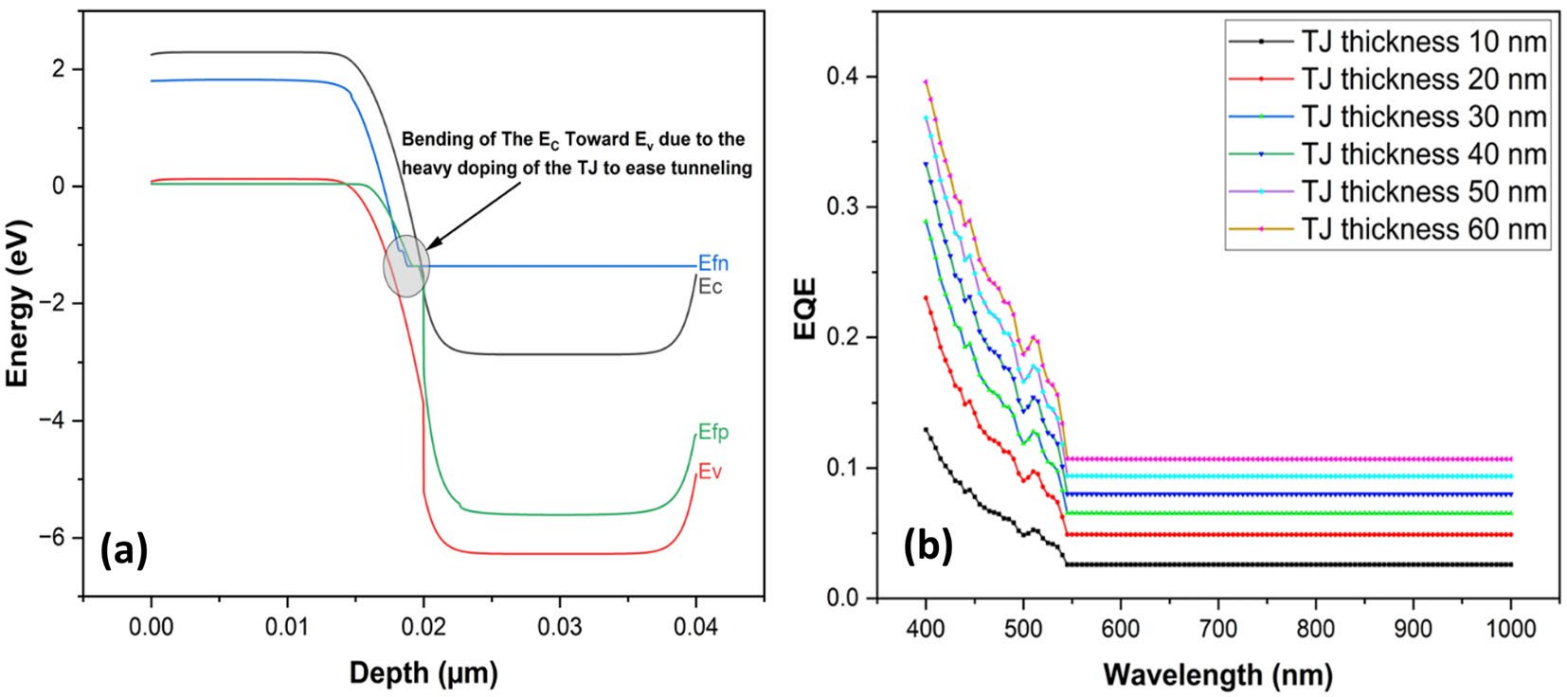
(**a**) Energy band diagram for the highly doped ZnTe/AZO tunneling junction and (**b**) Parasitic absorption of photons by the tunnel diode (TJ) at different thicknesses.

An effective ohmic tunnel diode is necessary for a monolithically connected tandem device to function properly^36^. A suitable tunnel diode, or a tunnel junction (TJ), can optically and electrically connect different sub-cells with minimal loss^37^. Trap-assisted and intra-band tunneling models are incorporated into wxAMPS for tunneling^38^. For CdTe/Si dual-junction solar cells, an effective tunnel junction is still being researched. In this work, a highly doped p++ zinc telluride (ZnTe) and n++ aluminum doped zinc oxide (AZO) (ZnTe/AZO) is proposed as TJ to connect the two subcells. The doping concentrations are 10^20^ cm^−3^ and 10^19^ cm^−3^ for ZnTe and AZO, respectively^39^. These high doping concentrations resulted in significant bending of the Ec of ZnTe towards the Ev of AZO to ease non-local band-to-band tunneling between the top and the bottom cells, as seen in Fig. 3a, and this will help in more carrier collection in the tandem solar cell^40^. Figure 3b shows that the wide bandgap materials used for the tunnel diode absorb some photons in the higher energy range (400–550 nm wavelength). The 10 nm thickness tunnel diode has the lowest absorption in this range of about 10%, and the TJ, having 60 nm thickness, has the highest absorption of around 40% at a wavelength of 400 nm. Increasing the thickness to 70 nm of the TJ caused the simulation return error, probably due to the high recombination of the generated carriers, making them go through a longer diffusion length before being collected. Proper TJ junction design is required using suitable materials to achieve an efficient CdTe/Si tandem design.

### Effect of defect density concentrations at various defect energy levels

**Figure 4:**
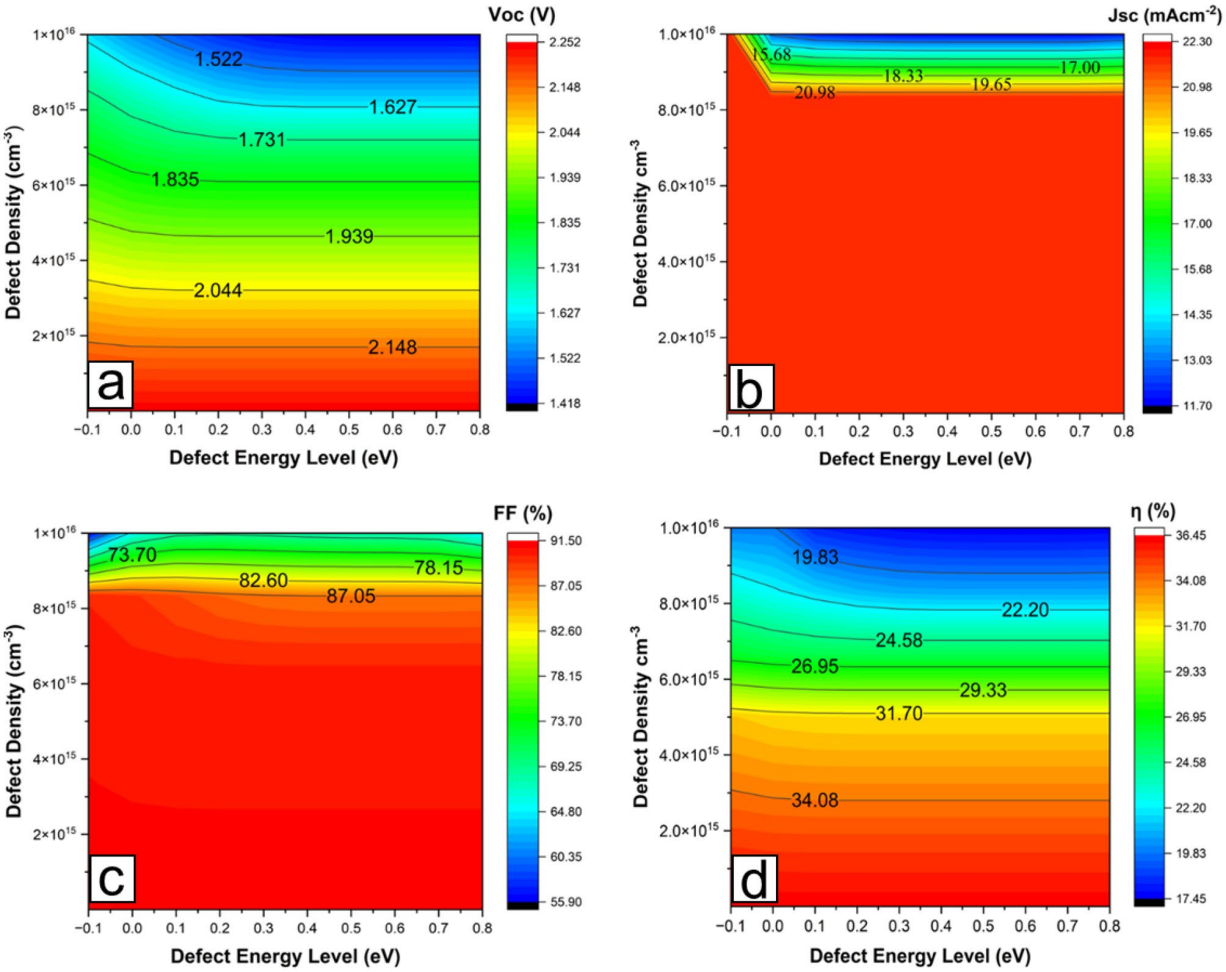
Contour plots for the various defect concentrations at different energy levels with (**a**) Open circuit voltage, Voc, (**b**) Short circuit current density, Jsc, (**c**) Fill factor, FF and (**d**) Efficiency, ƞ.

Defect states are key in determining the overall performance of a solar cell. These states, usually present in the material’s bandgap, cause undesirable trapping or scattering of mobile charge carriers, mostly lowering the solar cell’s open circuit voltage (Voc) and fill factor (FF). Depending on the position of the defect energy level, they could be shallow, deep level (mid-gap), or intermediate defects^41^. Figure 4a–d shows contour plots for the various defect concentrations at different energy levels. In this study, the defects are chosen to be located at different energy levels below the conduction band of the CdTe layer to cover these three different types of defect energy levels (E_t_). The effect of defect density at different energy levels on the device performance parameters of CdTe/Si dual-junction solar cells is reported.

The acceptor defect concentration of the CdS window layer is fixed at 1 × 10^14^ cm^−3^, while the donor defect concentration of the CdTe absorber layer is varied from 1 × 10^10^ cm^−3^ to 1 × 10^16^ cm^−3^. The defect introduced has a Gaussian distribution with a characteristic energy of 0.1 eV. In all the four output parameters extracted, poor Voc response is more pronounced due to the effect of the acceptor defect density. For the donor defect density close to the valence band, at defect energy levels between − 0.1 and 0.2 eV, there is an increase in Voc and Jsc. This could be due to the donor defects contributing to the mobile charge carriers in the valence band. While moving from defect energy levels of 0.2 eV to 0.8 eV, a considerable decrease in Voc was observed starting from a defect concentration of about 2 × 10^15^ cm^−3^. The Jsc deterioration becomes more noticeable from a defect density concentration of about 1 × 10^16^ cm^−3^ at all E_t_ above the valence band. This is clearly due to the recombination rate surge, which reduces the carrier lifetime^42^. Poor FF has resulted at a lower E_t_ than higher ones toward the middle of the band gap. The overall efficiency is affected more by a defect concentration of about 4 × 10^15^ cm^−3^ and above E_t_. The defect range allowable for a thickness of 300 nm CdTe absorber layer in this simulation is 10^14^ to 10^15^ cm^−3^ at all E_t_. A similar result was reported before^43^.

### Impact of defect density at varying thicknesses of the CdTe top cell absorber layer

**Figure 5:**
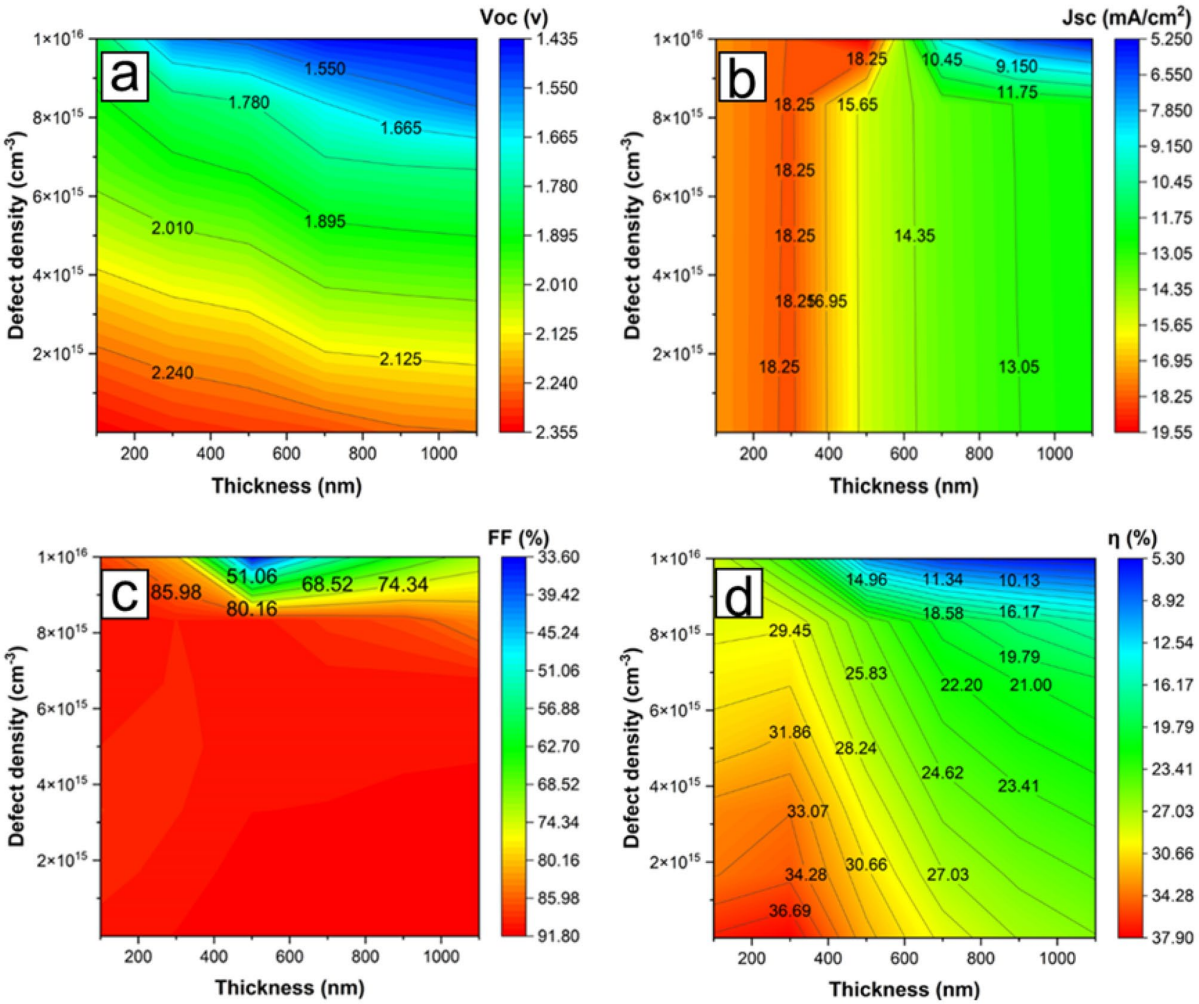
Contour plot for the various defect concentrations at different thicknesses of the CdTe absorber layer with (**a**) Open circuit voltage, Voc (**b**) Short circuit current density, Jsc (**c**) Fill factor, FF and (**d**) Efficiency, ƞ.

In a monolithically connected dual junction solar cell, optimizing the device’s current density by thinning the top cell absorber layer thickness, as performed in Fig. 2a–d, is imperative. Defects may be introduced in this layer because of the thinning and other factors, such as the device fabrication process, exposure to external factors, material defects, etc., which can all affect the performance of the device in a significant way^35^. To help understand these defects and how to mitigate their impact during the design and fabrication of the device, concentrations of donor defect ranging from 1 × 10^10^ to 1 × 10^16^ cm^−3^ are introduced in the CdTe top layer at different CdTe thicknesses varied from 100 to 1100 nm. The results obtained reveal that the Jsc is optimum from a thickness of around 260 nm until around 300 nm. This is clearly due to the current matching between the bottom and the top cell while varying the thickness of the CdTe layer. The Jsc is primarily affected by the thickness of the CdTe absorber layer regardless of defect density concentrations. The response to the effect of the defect density is more pronounced on Voc, especially at a defect density above 10^15^ cm^−3^, but reduces with the thickness of the CdTe layer, as shown in Fig. 5a,b. This may not be unconnected with the defects due to pinholes becoming less as the CdTe layer becomes thicker. The FF is not affected much by the thickness of the CdTe than the defect concentration, as shown in Fig. 5c. Overall, an optimum efficiency was obtained for this design at a defect concentration of around 10^15^ cm^−3^ at a CdTe thickness of 300 nm, as indicated by Fig. 5d.

### Top and bottom subcells bandgap matching

**Figure 6:**
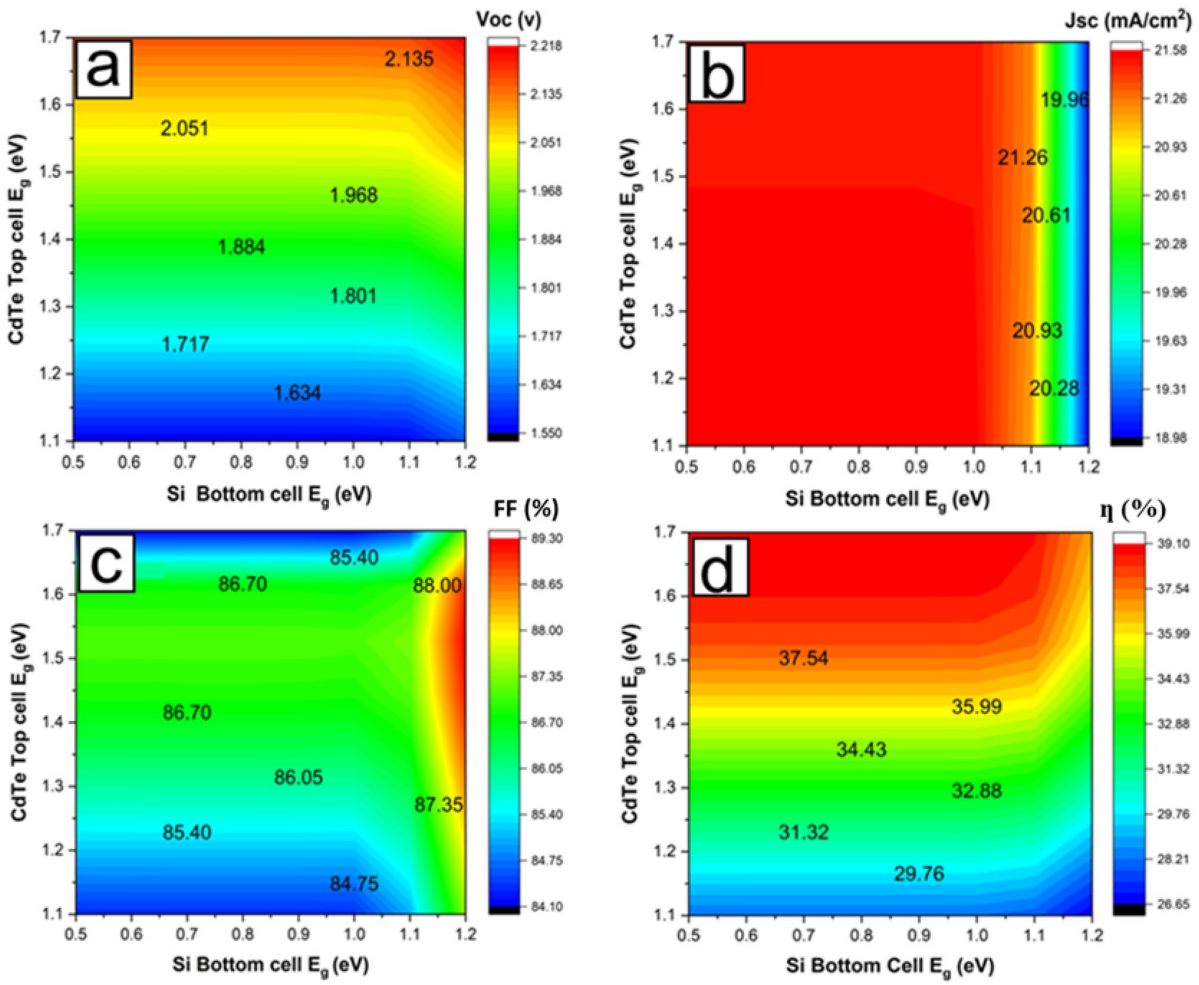
Variations of CdTe Top-cell bandgap and the bottom Si cell bandgap with a) Open circuit voltage, Voc (b) Short circuit current density, Jsc (c) Fill factor, FF and (d) Efficiency, ƞ.

To validate the advantage of using CdTe and its alloys having a bandgap between 1.5 to 1.7 eV as the top absorber materials for Si solar cells with a band gap of around 1.12 eV, the energy bandgaps of both the top and bottom absorber layers were varied within between 1.1–1.7 eV for CdTe and 0.5–1.2 eV for Si to obtain various values for the Voc, Jsc, FF and ƞ in the design of CdTe/Si device. Si band gap does not usually vary as assumed in this simulation. Still, we varied it to demonstrate the 3D heat map of CdTe bandgap variations, which may also be applied to other potential bottom cell materials with different bandgaps from Si. Figures 6a–d show an optimum performance with a bandgap combination between 1.45–1.7 eV of the CdTe top layer with a Si bandgap of about 1.1 eV. The Voc of the simulated tandem device is mainly affected by CdTe E_g_. Therefore, turning the bandgap value will significantly improve the Voc. As explained earlier, this can be achieved by doping the CdTe with elements such as zinc (Zn) and Magnesium (Mg). However, the Jsc is constrained by the Si E_g_. Finally, the FF is less affected by either Si or CdTe E_g_. The best E_g_ combination is obtained at CdTe E_g_ > 1.4 eV and Si E_g_ of 1.1 eV. This result is consistent with the theoretical results^44–47^. This makes CdTe and its alloys a good choice for bandgap combination with a Si bottom cell as a tandem solar cell device.

## Conclusion

This work has elucidated and optimized the effect of defects at different defect energy levels and thicknesses of the CdTe top cell in a proposed CdTe/Si dual-junction tandem solar cell. The impact of defect concentration is studied against the thickness of the CdTe absorber layer. Validation of the bandgap combinations between CdTe and Si in a tandem form has also been carried out. The results show that a CdTe defect density of 10^14^ and 10^15^ cm^−3^ is tolerable for the designed CdTe/Si solar cell, which exists within a spectrum of the studied defect energy levels. The variations of defect concentrations against the thickness of the CdTe top absorber layer indicate that the open circuit voltage (Voc), short circuit current density (Jsc), and efficiency (ƞ) are more affected by the defect density at higher CdTe thickness. At the same time, the Fill factor (FF) is more affected by the defect density than the thickness. An acceptable defect density of 10^15^ cm^−3^ at a CdTe top layer thickness of 300 nm is obtained from this work. Though it is difficult to deposit defect-free CdTe at 300 nm of thickness due to the lattice mismatch issue, careful optimization of the deposition method, deposition parameters, post-deposition treatment, and matched buffer layers will be required for the realization of efficient monolithically connected CdTe/Si tandem solar cell. The bandgap variation shows a good result for a CdTe bandgap ranging from 1.45 to 1.7 eV in tandem with a Si bandgap of about 1.1 eV. This work reveals that it is important for defects and the bandgaps of absorber layers to be optimized for the realization of efficient CdTe/Si dual junction tandem solar cells.

## Data Availability

The datasets used and analyzed during the current study are available from the corresponding author upon reasonable request.
